# Association between Subjective Cognitive Complaints and Sleep Disturbance among Community-Dwelling Elderly Individuals in Japan

**DOI:** 10.3390/healthcare12131245

**Published:** 2024-06-22

**Authors:** Akio Goda, Hideki Nakano, Yuki Kikuchi, Kohei Mori, Nozomi Mitsumaru, Shin Murata

**Affiliations:** 1 Hokuriku University Well-Being Research Team, Department of Physical Therapy, Faculty of Health and Medical Science, Hokuriku University, Kanazawa 920-1180, Japan; 2 Department of Physical Therapy, Faculty of Health Sciences, Kyoto Tachibana University, Kyoto 607-8175, Japan; 3 Faculty of Allied Health Sciences, Kansai University of Welfare Sciences, Kashiwara 582-0026, Japan; 4 Kusukinomori Co., Ltd., Imari 848-0027, Japan

**Keywords:** aged, community-dwelling, dementia, east Asian people, risk, sleep deprivation, smoking, subjective cognitive complaints

## Abstract

Subjective cognitive complaints (SCCs) are a crucial modifiable risk factor for dementia. There is increasing interest in the association between SCC and sleep disturbance; however, the effects of sleep disturbance on SCC development among community-dwelling elderly individuals in Japan remain unclear. We aimed to cross-sectionally investigate the association between SCC and sleep disturbance, with adjustment for multiple factors related to cognitive decline, among 241 community-dwelling elderly persons without cognitive impairment. The measures were SCCs (Kihon Checklist-Cognitive Function, KCL-CF), sleep disturbance (Japanese version of the Athens Insomnia Scale, AIS-J), general cognitive function (Mini-Mental State Examination), and depressive symptoms (five-item version of the Geriatric Depression Scale [GDS-5]). The following data were collected: sex, age, educational history, whether the participants had visited a medical institution for diseases (hypertension, diabetes, hyperlipidemia, heart disease), and the presence/absence of established risk factors (hearing loss, history of head injury, drinking habits, smoking habits, social isolation, and physical inactivity and activity). Based on the KCL-CF, 96 and 145 participants were considered to have and lack SCCs, respectively. On logistic regression analysis, the AIS-J score and smoking history were significantly associated with SCCs. Our findings suggest that sleep disturbance is associated with SCC development among community-dwelling elderly people in Japan. Evaluating and managing sleep disturbances can be important in preventing SCCs and dementia.

## 1. Introduction

With population aging, dementia has become a major public health concern [1]. The global prevalence of dementia is projected to nearly triple in the coming decades, with an estimated 150 million individuals affected by 2050 [2]. Dementia imposes a huge burden on individuals, families, and society as a whole [3]. Currently, dementia remains an incurable disease; accordingly, early interventions for preventing the onset of dementia are crucial [4].

Alzheimer’s disease (AD) is the leading cause of dementia, accounting for 60–80% of all dementia cases [5]. According to a proposed framework, the stages of dementia begin with a healthy state, progressing to subjective cognitive complaints (SCCs) as a preclinical sign, mild cognitive impairment (MCI), and finally AD [6]. SCCs encompass daily memory issues and related cognitive concerns expressed by individuals, regardless of the presence of objective evidence of cognitive impairment; they are common across all age demographics [7]. However, SCCs strongly influence the development of MCI [8] and AD [9]. Moreover, pathological changes in brain tissue begin during the SCC stage, decades prior to AD onset [10,11]. Accordingly, establishing preventive and therapeutic interventions focusing on SCCs is imperative for delaying the onset of dementia in the future [12].

Several risk factors at various life stages exist for dementia development and addressing these modifiable risk factors is important to prevent dementia onset [13]. Recently, sleep disturbance has been highlighted as among the modifiable risk factors for dementia development [14]. Sleep is crucially involved in brain function and health; accordingly, sleep insufficiency or impairment can lead to various cognitive problems [15]. To this point, sleep problems may influence the development of AD [16], MCI [17], and SCCs [18].

However, studies on the association between sleep and SCCs in Japan remain limited [19]. Additionally, regional and racial differences in SCC onset may exist [20]. Thus, elucidating the impact of sleep on SCC development in the Japanese population is important. Furthermore, previous Japanese studies have failed to appropriately adjust for multiple factors associated with dementia development, which compromises the reliability of their findings. Therefore, we aimed to examine the influence of sleep disturbances on SCC development among community-dwelling elderly people in Japan, with adjustment for multiple factors related to cognitive decline. Our findings could inform the early detection of the risk of SCC development and the establishment of preventive measures and intervention programs against the future onset of dementia.

## 2. Materials and Methods

This cross-sectional survey of community-dwelling elderly people living in Japan was conducted between September 2021 and September 2022. Participants were recruited through flyers distributed annually from June to August in Imari City, Saga Prefecture between 2021 and 2022. These flyers clearly indicated that participation was unpaid. Data regarding the participants’ sex, age, and body mass index (BMI) were collected. Moreover, the participants underwent the Mini-Mental State Examination (MMSE) as a test of general cognitive function. The eligibility criteria for this study were as follows: no previous participation in this study; age ≥65 years; and the absence of cognitive decline, with an MMSE score ≥ 24 [21]. Meanwhile, those with a history of psychiatric or cerebrovascular disease and those who were incapable of completing all the measurement items were excluded.

**Figure 1:**
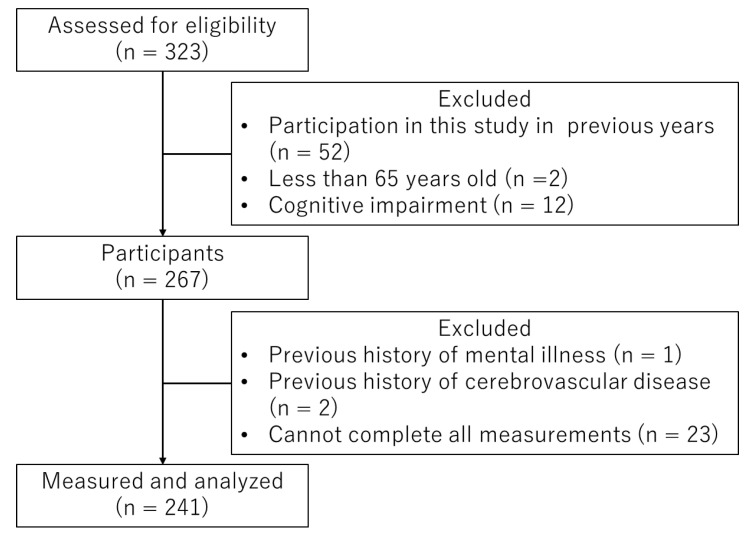
Flowchart of the selection of the study participants.

Finally, this study included 241 participants (39 males and 202 females; mean age, 77.3 ± 5.9 years) (Figure 1). Written informed consent was obtained from each participant prior to participation. This study was conducted in accordance with the Declaration of Helsinki and approved by the Ethics Committee of Kyoto Tachibana University (accession nos. 18–26).

In addition to general cognitive function, several other parameters were assessed. SCCs were assessed using the Kihon Checklist-Cognitive Function (KCL-CF). The severity of insomnia symptoms was assessed using the Japanese version of the Athens Insomnia Scale (AIS-J). The severity of depressive symptoms was assessed using the five-item version of the Geriatric Depression Scale (GDS-5). Moreover, the researcher conducted an oral interview with the participants regarding the presence or absence of risk factors for the development of dementia.

### 2.1. General Cognitive Functioning (MMSE)

General cognitive function was assessed using the MMSE [22], which is a short test that is widely used to assess cognitive function. This screening tool, which covers 11 domains including attention, executive functioning, agnosia, language, memory, orientation, praxis, prosody, thought content, thought processes, and visuospatial proficiency, has been shown to be effective [23] and reliable [21]. The participants’ cognitive performance was evaluated based on the total MMSE scores (range: 0–30), which covered all the domains measured.

### 2.2. SCC (KCL-CF)

The SCC status was ascertained through an interview. The participants were asked three questions that comprised the cognitive function domain of the KCL-CF, which is a self-report questionnaire designed to assess frailty that has established validity [24,25] and reliability [26]. These questions included “Do your family or your friends point out your memory loss?”, “Do you make a call by looking up phone numbers?”, and “Do you find yourself not knowing today’s date? [26]”. The KCL-CF has been previously used as a measure of subjective memory complaints [27] and self-reported cognitive decline [28]. However, we used the KCL-CF as a measure of the participants’ SCC status [29]. Each positive response (i.e., a response suggestive of SCCs) was assigned 1 point, a KCL-CF score (range: 0–3) was calculated, and the participants with a KCL-CF score ≥ 1 were considered to have SCCs [29].

### 2.3. Insomnia Symptoms (AIS-J)

The severity of insomnia symptoms was assessed using the AIS-J [30]. The AIS-J comprises eight items assessing insomnia symptoms within the past month (including (1) sleep initiation; (2) awakening during the night; (3) early morning awakening; (4) total sleep duration; (5) overall quality of life; (6) problems with sense of well-being; (7) overall functioning; and (8) daytime sleepiness). Each AIS-J item is rated on a four-point scale (0 = no problems at all; 1 = some problems; 2 = significant problems; 3 = very significant problems), with a higher total score (range: 0–24) indicating more severe insomnia. Based on the AIS-J score, the severity of insomnia was classified as pathological insomnia (6–24), nocturnal insomnia (4–5), and no insomnia (0–3). The validity [30] and reliability [31] of the AIS-J have already been reported.

### 2.4. Depressive Symptoms (GDS-5)

Depressive symptoms were assessed using the GDS-5 (a simplified version of the GDS [32]). This is a self-reported screening questionnaire designed to focus on the characteristic manifestations of depressive symptoms in the elderly population. This tool has been shown to be valid and reliable [33]. The GDS-5 comprises five questions [32], including (1) Are you satisfied with your life?; (2) Do you often get bored?; (3) Do you often feel helpless?; (4) Do you prefer to stay at home, rather than going out and doing new things?; and (5) Do you feel pretty worthless the way you are now?, which are answered on a yes/no basis. Each positive response (i.e., indicating depressive symptoms) receives one point, with the total score of all the items (range: 0–5) indicating the overall symptom severity.

### 2.5. Risk Factors for Dementia Development

We performed interviews for the following risk factors for developing dementia: sex [34]; age [35]; educational level [36]; a history of hypertension [37], diabetes [38], hyperlipidemia [39], and cardiovascular disease [40]; the presence of known risk factors such as hearing loss [41], a history of a head injury [42], past or current alcoholic drinking habits [43], and past or current smoking habits [44]; social isolation (no individual to interact with (in person, by phone, or through email) at least once per week [except for family members who live with you]) [45]; and physical inactivity (not exercising for ≥30 min a day, 2 days a week for at least 1 year [46]).

### 2.6. Sample Size Calculation

The sample size was calculated using G*Power software version 3.1.9.7. Based on a previous report [19], the prevalence of SCCs in the group with sleep disturbances was estimated to be 63%. Based on the statistical power of 80% and a level of significance set at 5%, we calculated the total sample size to be 193 participants. The sample size was increased by 67% to 323 patients to account for loss to the adaptation of the exclusion criteria.

### 2.7. Statistical Analysis

First, we checked the distribution normality of all the demographic and rating variables using the Shapiro–Wilk test. Next, the variables were compared between the participants with and without SCCs using the independent samples *t*-test (age), the Mann–Whitney U test (BMI, educational history, AIS-J, GDS-5, and MMSE), Fisher’s exact test (insomnia severity ranked by AIS-J), and the χ^2^ test (other variables). Finally, we examined the significance of the association between SCC occurrence and each variable using logistic regression analysis (forced entry) adapting a model wherein the presence of SCCs was the dependent variable (1: presence, 0: absence) and all other items were independent variables. All the statistical analyses were performed using SPSS Statistics software (version 26, IBM, New York, NY, USA), with a significance level of 5%.

## 3. Results

In our study, 145 participants had a KCL-CF score of 0, 75 had a score of 1, 19 had a score of 2, and 2 had a score of 3. Accordingly, 39.8% of the participants were evaluated to have SCCs (KCL-CF score ≥ 1). Based on the AIS-J, the insomnia severity was determined to be pathological insomnia (6–24 points) in 34 (14.1%) participants, nocturnal insomnia (4–5 points) in 48 (19.9%) participants, and no insomnia (0–3 points) in 159 (66.0%) participants. Fisher’s exact test results revealed that the participants with SCCs had a significantly higher percentage of severe insomnia (Table 1; *p* = 0.0004).

Table 2 shows the between-group comparisons of the basic attributes and various measurement items. Compared with the participants without SCCs, those with SCCs were significantly older, had a lower educational level, had higher AIS-J scores, had higher GDS-5 scores, had lower MMSE scores, and a higher proportion of participants had past or current smoking habits (*p* < 0.05).

Logistic regression analysis (forced entry) was performed with the presence of SCCs as the dependent variable (Table 3). The results indicated that the AIS-J (OR, 1.16; 95% CI, 1.03–1.31; *p* < 0.05) and having a past or current smoking habit (OR, 3.48, 95% CI, 1.24–9.70, *p* < 0.05) remained significant explanatory variables after adjusting for various risk factors for dementia.

## 4. Discussion

In this study, we hypothesized that insomnia symptoms would be associated with the incidence of SCCs among community-dwelling elderly people in Japan. Our findings indicated that the patients with SCCs were significantly older, had a lower educational level, had more severe symptoms of insomnia (AIS-J) and depression (GDS-5), had lower general cognitive function (MMSE), and had a higher proportion of past or current smoking habits than the patients without SCCs. Furthermore, logistic regression analysis with adjustment for various risk factors for dementia demonstrated that severe insomnia was an independent factor associated with the incidence of SCCs.

In our study, the incidence of SCCs (KCL-CF score ≥ 1) was 39.8%, which is higher than previously reported values among community-dwelling older adults in Japan, including rates of 32.5% [28], 34.5% [47], 34.9% [29], 35.4% [48], and 37.6% [49]. This may be attributed to an increase in the incidence of SCCs with age [50]. Compared with participants in previous studies (mean age: 73.3–75.9 years) [28,29,47,48,49], our participants were slightly older (mean age, 77.3 ± 5.9 years), which may have resulted in a higher incidence of SCCs. Taken together, the incidence rate of SCCs in our study was within the expected range based on previously reported values.

Compared with the participants without SCCs, those with SCCs were older, had a lower educational level, had more severe symptoms of insomnia (AIS-J) and depression (GDS-5), had lower general cognitive functioning (MMSE), and a higher proportion of them had past or current smoking habits (Table 2). Furthermore, these items were significantly associated with the presence of SCCs in the single regression analysis (Table 3). Consistent with our findings, previous studies have reported significant differences in the following factors among older adults with SCCs: age [50,51], educational history [52,53], insomnia symptoms [54], depressive symptoms [18], general cognitive functioning [55], and current [56,57] and past [58] smoking habits. Taken together, these findings corroborate the demographic and neurophysiological characteristics of community-dwelling elderly individuals with SCCs in Japan.

Logistic regression analysis with adjustment for several risk factors for dementia revealed that the factors associated with the incidence of SCCs were the severity of insomnia and having a past or current smoking habit (Table 3). Previous studies have reported that insomnia is associated with the development of SCCs [51,54,59,60,61,62,63,64,65,66]. This could be attributed to the disruption of functional connectivity of the major resting-state networks in patients with insomnia [67]. These abnormalities in functional connectivity are associated with SCC severity [68,69,70] and may mediate the effect of sleep disturbances on SCC development. Another contributing factor is the presence of insomnia-induced neuropsychiatric symptoms (NPS), including depression and anxiety [71,72]. The presence of these NPS influences the development of SCCs [73] and may explain the high incidence of SCCs among individuals with insomnia. Furthermore, taking sleeping pills has been reported to be associated with SCCs [74]. Individuals with insomnia symptoms frequently use sleeping pills [75], which may contribute to SCC development. Regarding smoking habits, current and past smoking habits each have an effect on SCC development [56]. Moreover, the risk of SCCs is positively correlated with cumulative exposure to smoking among current or past smokers [58]. Our findings are consistent with these previous reports. The proposed mechanisms through which smoking affects cognitive function include impairment of endothelial function, inhibition of brain oxygen supply, increased oxidative stress, altered mitochondrial energy metabolism, decreased synaptic connectivity, and metabolic enzymes involved in amyloid-β and tau proteins [76]. To this point, SCCs were more likely to occur among the participants who presented with these factors.

Our findings indicated that the development of SCCs in community-dwelling elderly individuals was influenced by insomnia and smoking habits. These factors are both modifiable, treatable, and may be targeted to prevent the development of SCCs among community-dwelling elderly persons.

To this point, the following methods can be applied to the prevention and management of SCCs among community-dwelling elderly persons in Japan:

Addressing sociodemographic conditions (low income, unemployment), poor health behaviors (alcohol consumption, diabetes, hypertension, inactivity, obesity), and mental health conditions (poor subjective health status, stress, depressive symptoms) [77] associated with the development of insomnia and establishing interventions (e.g., meditation, music appreciation) [78] to improve insomnia symptoms

Reducing exposure to smoking and passive smoking by providing health education and environmental improvement recommendations [58].

This study has several limitations. First, given the cross-sectional design, we could not establish a causal relationship. Longitudinal studies are warranted to clarify the relationship between the changes in each indicator and SCC development. Second, we could not collect detailed information regarding the participants’ objective sleep assessment and medication status. Future studies are warranted to provide additional details. Finally, our participants were relatively healthy. It remains unclear whether our findings can be applied to a population with poorer health.

Nonetheless, this study is significant because it suggests that modifiable factors, namely insomnia and smoking habits, may influence SCC development among community-dwelling elderly in Japan.

## 5. Conclusions

This study examined the association between SCC prevalence and sleep disorders among community-dwelling elderly in Japan. Our findings indicated that SCC prevalence was associated with the severity of sleep disorders and smoking habits. Therefore, geriatricians need to implement measures that target modifiable factors, such as improving sleep disorders and teaching smoking cessation. Despite some limitations, our findings may help healthcare professionals in the field of geriatrics to design and implement more effective intervention programs for preventing dementia in the elderly population.

## Figures and Tables

**Table 1:** Group comparison of insomnia severity by Fisher’s exact test.

Variable		SCC	Non-SCC
AIS-J	No insomnia	49	110
	Nocturnal insomnia	27	21
	Pathological insomnia	20	14

SCC group: KCL-CF score ≥ 1; non-SCC group: KCL-CF score = 0; no insomnia group: AIS-J score = 0–3; nocturnal insomnia group: AIS-J score = 4–5; pathological insomnia group: AIS-J score = 6–24; SCCs, subjective cognitive complaints; AIS-J J, Japanese version of the Athens Insomnia Scale.

**Table 2:** Between-group comparisons of the basic attributes and various measurement items.

Variable		SCC	Non-SCC	*p*-Value
		(n = 96)	(n = 145)	
Attribute	Age (yr) ^☨^	78.25 ± 5.95	76.70 ± 5.80	0.05
	Sex: Male/Female (*n*)	19/77	20/125	0.22
	BMI (kg/m^2^) *	22.42 ± 3.43	22.48 ± 3.29	0.72
	Educational history (yr) *	11.178 ± 2.23	11.91 ± 2.41	0.02
Mental and physical indicator	AIS-J (score) *	3.70 ± 3.09	2.41 ± 2.20	0.001
	GDS-5 (score) *	0.95 ± 1.20	0.52 ± 0.84	0.004
	MMSE (score) *	27.88 ± 1.95	28.39 ± 1.85	0.02
Medical visits due to disease	Hypertension: yes/no (*n*)	45/51	52/93	0.09
	Diabetes: yes/no (*n*)	10/86	16/129	0.88
	Hyperlipidemia: yes/no (*n*)	13/83	34/111	0.06
	Cerebrovascular disease: yes/no (*n*)	5/91	5/140	0.50
Presence of dementia risk	Hearing loss: yes/no (*n*)	28/68	32/113	0.21
	History of head injury: yes/no (*n*)	8/88	6/139	0.17
	Past or current drinking habits: yes/no (*n*)	27/69	39/106	0.83
	Past or current smoking habits: yes/no (*n*)	23/73	13/132	0.001
	Social isolation: yes/no (*n*)	9/87	20/125	0.30
	Physical inactivity: yes/no (*n*)	28/67	68/114	0.17

Data are presented as mean ± standard deviation; SCC group: KCL-CF score ≥ 1; non-SCC group: KCL-CF score = 0; χ^2^ test; ^☨^: *t*-test using independent samples; *: Mann–Whitney U test; SCCs, subjective cognitive complaints; *n*, number; yr, year; BMI, body mass index; kg, kilogram; m, meter; AIS-J, Japanese version of the Athens Insomnia Scale; GDS-5, five-item version of the Geriatric Depression Scale; MMSE, Mini-Mental State Examination.

**Table 3:** Logistic regression analysis with the presence or absence of SCCs as the dependent variable.

Variable			Univariate Analysis				Multivariate Analysis			
				95% CI for OR				95% CI for OR		
			OR	Lower	Upper	*p*	OR	Lower	Upper	*p*
Attribute	Age (yr)		1.05	1.00	1.09	0.05	1.03	0.97	1.09	0.37
	Sex	Female	0.65	0.33	1.29	0.22	0.68	0.21	2.23	0.53
		Male	1.00				1.00			
	BMI (kg/m^2^)		0.99	0.92	1.07	0.89	0.97	0.88	1.06	0.46
	Educational history (yr)		0.87	0.78	0.98	0.02	0.87	0.75	1.00	0.06
Mental and physical indicator	AIS-J (score)		1.21	1.09	1.34	0.001	1.16	1.03	1.31	0.02
	GDS-5 (score)		1.52	1.17	1.98	0.004	1.33	0.98	1.82	0.07
	MMSE (score)		0.87	0.76	0.99	0.04	0.91	0.77	1.07	0.25
Medical visits due to disease	Hypertension	Yes	1.58	0.93	2.67	0.09	1.68	0.90	3.16	0.11
		No	1.00				1.00			
	Diabetes	Yes	0.94	0.41	2.16	0.88	0.88	0.33	2.35	0.80
		No	1.00				1.00			
	Hyperlipidemia	Yes	0.51	0.25	1.03	0.06	0.59	0.27	1.28	0.18
		No	1.00				1.00			
	Cerebrovascular disease	Yes	1.54	0.43	5.46	0.51	1.26	0.28	5.66	0.77
		No	1.00				1.00			
Presence of dementia risk	Hearing loss	Yes	1.45	0.81	2.62	0.21	0.99	0.48	2.03	0.98
		No	1.00				1.00			
	History of head injury	Yes	2.11	0.71	6.27	0.18	2.42	0.65	8.99	0.19
		No	1.00				1.00			
	Past or current drinking habits	Yes	1.06	0.60	1.89	0.83	0.80	0.37	1.75	0.58
		No	1.00				1.00			
	Past or current smoking habits	Yes	3.20	1.53	6.69	0.001	3.48	1.24	9.70	0.02
		No	1.00				1.00			
	Social isolation	Yes	0.65	0.28	1.49	0.30	0.48	0.18	1.23	0.12
		No	1.00				1.00			
	Physical inactivity	Yes	1.51	0.84	2.74	0.17	1.55	0.76	3.18	0.23
		No	1.00				1.00			

SCCs, subjective cognitive complaints; OR, odds ratio; CI, confidence interval; yr, year; BMI, body mass index; kg, kilogram; m, meter; AIS-J, Japanese version of the Athens Insomnia Scale; GDS-5, five-item version of the Geriatric Depression Scale; MMSE, Mini-Mental State Examination.

## Data Availability

Qualified researchers can obtain the data presented in this study from the corresponding author.
